# Photofunctions in Hybrid Systems of Schiff Base Metal Complexes and Metal or Semiconductor (Nano)Materials

**DOI:** 10.3390/ijms231710005

**Published:** 2022-09-02

**Authors:** Takashiro Akitsu, Barbara Miroslaw, Shanmugavel Sudarsan

**Affiliations:** 1 Department of Chemistry, Faculty of Science, Tokyo University of Science, 1-3 Kagurazaka, Shinjuku-ku, Tokyo 162-8601, Japan; 2 Department of General and Coordination Chemistry and Crystallography, Institute of Chemical Sciences, Faculty of Chemistry, Maria Curie-Sklodowska University in Lublin, Pl. Marii Curie-Sklodowskiej 3, 20-031 Lublin, Poland; 3 Department of Chemistry, Rajalakshmi Engineering College (Autonomous), Thandalam 602 105, Tamilnadu, India

**Keywords:** photofunction, Schiff base, metal complex, photocatalysts, semiconductors, nanomaterials, DSSC (dye sensitized solar cell), hybrid materials

## Abstract

Composite materials very often provide new catalytic, optical or other physicochemical properties not observed for each component separately. Photofunctions in hybrid systems are an interesting topic of great importance for industry. This review presents the recent advances, trends and possible applications of photofunctions of hybrid systems composed of Schiff base metal complexes and metal or semiconductor (nano)materials. We focus on photocatalysis, sensitization in solar cells (DSSC—dye sensitized solar cell), ligand-induced chirality and applications in environmental protection for Cr(VI) to Cr(III) reduction, in cosmetology as sunscreens, in real-time visualization of cellular processes, in bio-labeling, and in light activated prodrug applications.

## 1. Introduction

Photocatalysis seems to be the ultimate solution for the global energy crisis. However, there are still missing large scale sustainable solar energy storage systems. One of the emerging problems is the lack of efficient, cost-effective and, preferably, non-toxic photocatalyst materials for large scale applications [1,2,3,4,5,6,7,8]. The answer to this need may be among others novel hybrid materials built of Schiff base metal complexes combined with metal or semiconductor (nano)materials such as gold or TiO_2_ nanoparticles, which exhibit a wide range of photofunctions and may find applications also in other scientific and industrial branches.

In general, Schiff bases and their metal complexes are multipurpose compounds produced from the condensation of amines with carbonyl compounds and are extensively utilized in many research and industrial applications. These compounds are often utilized alone or for a preparation of various hybrid materials, such as, for example, supramolecular elastomers with imine-functionalized polysiloxanes [9], conducting metallopolymers for electrochemical sensing [10], hybrid SiO_2_ nanoparticles with flame retardant and antibacterial properties [11] or recently reported cocrystals of a Schiff base with lead iodide perovskite showing photo-triggered ferroelectricity [12].

There are many original papers, as well as excellent reviews on the current state of knowledge in chemistry of such compounds concerning their synthesis methods, crystal structure and useful properties including bioactive, magnetic, catalytic, electrochemical and photoluminescent ones [10,13,14,15,16,17,18,19,20]. The current review focuses on very interesting photofunctions observed in hybrid systems built of Schiff base metal complexes and various materials, such as metal or semiconductor nanoparticles, as well as polymer matrices. These composite materials provide new catalytic and optical properties not observed for each system separately and give new possibilities to use them for solar cell materials as sensitizers or co-sensitizers, in optoelectronic devices, in catalysis as for example Cr(VI) reductors and many more. The current review shows the recent advances, trends and possible applications of photofunctions of such hybrid systems.

## 2. Photocatalysis—UV-Induced Reduction of Cu(II) Complexes by TiO_2_

Takeshita et al. [21,22,23,24,25,26] created a complex system of a tetracoordinated planar chiral Schiff base amino acid Cu(II) complex and a TiO_2_ photocatalyst. They evaluated the expression of electronic functions and the micro-optical electronic state by ultraviolet light irradiation. This photocatalytic system enabled the reduction of the Cu(II) ions under ultraviolet light in the presence of the TiO_2_ because of the intermolecular photoelectron transfer, which cannot be observed by the complex alone.

The dye sensitization was adjusted in this hybrid system by a special design of the ligand molecules of the chiral metal complexes. The aim of the research was to obtain the knowledge applicable to the solar cells. To develop such systems, several chiral Schiff base amino acid Cu(II) complexes were synthesized using various amino acids (valine, lysine, methionine, threonine, arginine, asparagine and phenylalanine) and salicylaldehyde derivatives.

**Figure 1:**
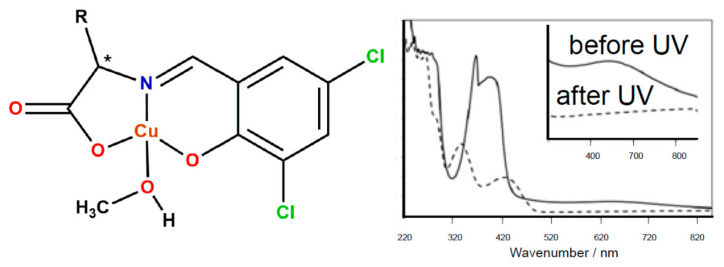
Schiff base Cu(II) complex (* denotes an asymmetric carbon atom) reduced in the presence of TiO_2_ after UV light irradiation and UV-Vis spectral changes associated with d-d transition of Cu(II) species before and after UV light irradiation [27].

As a development to the model catalyst system for the water splitting the reduction reaction of Schiff base Cu(II) complex to Cu(I) by TiO_2_ after ultraviolet light irradiation was studied by Nishizuru et al. [27]. The photoreduction reaction after irradiation with UV light resulted in the decrease of the absorption intensity of the d-d transition band near 650 nm, suggesting that the Cu(II) complex was reduced to the Cu(I) complex (Figure 1).

Kurata et al. [23] studied the mechanism of action of the hybrid systems composed of Cu(II) complexes and TiO_2_ microparticles affected by UV light and oxygen exposed. They synthesized a novel chiral threonine derived Schiff base Cu(II) complex having a biocompatible imidazole co-ligand, which was prone to be dissociated. The electrochemical studies showed that the Cu(II)/Cu(I) redox reaction in the presence of titanium oxide was reversible, regardless of exchanging the co-ligand to solvent molecule.

**Figure 2:**
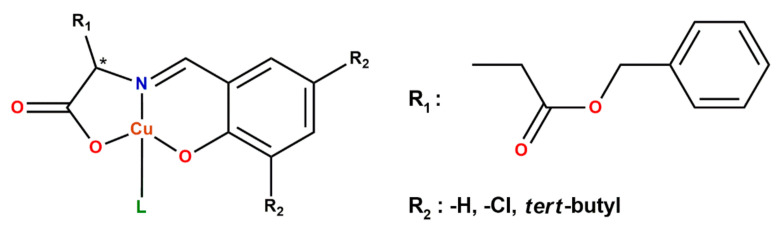
Ester type amino acid Schiff base Cu(II) complexes (* denotes an asymmetric carbon atom) [28].

Yoshida et al. [28] synthesized a novel chiral amino acid ester derivative Cu(II) complex in which the side chain carboxyl group of glutamate was esterified with a benzyl alcohol (Figure 2). It was investigated whether the photoinduced electron transfer (PET) will occur even with the presence of a large steric hindrance in the ligand. The reduction reaction of the Cu(II) complex proceeded by irradiation with ultraviolet light occurred eventually, in the TiO_2_ composite system; however, the irradiation with ultraviolet light did not change the absorption spectrum until the first 20 min. After that, an increase in the absorption peak due to the d-d transition was observed. This increase in absorption peak was the opposite of that of the non-ester complex.

## 3. Sensitization in Solar Cells (DSSC)

Dye-Sensitized-Solar-Cell (DSSC) is a promising approach in modern solar cell technology [29,30,31,32,33,34]. The efficiency of Ru complexes is around 10% and additionally the price of this noble metal is quite high; therefore, there is still need for better photosensitizers. Many research groups tried to replace ruthenium with other transition metal complexes for application in DSSC. Recently, Mahadevi and Sumathi published a mini review on the application of Schiff base and their metal complexes as photosensitizers [35]. The limitation of salen type complexes is that π-conjugation is difficult to be spread. Therefore, Yamaguchi et al. [36] synthesized a new group of binaphthyl Schiff base complexes of Cu(II), Ni(II), and Zn(II)) with carboxyl group to overcome this problem. The authors evaluated the complexes for application in DSSC by TD-DFT calculation and experimental analysis.

**Figure 3:**
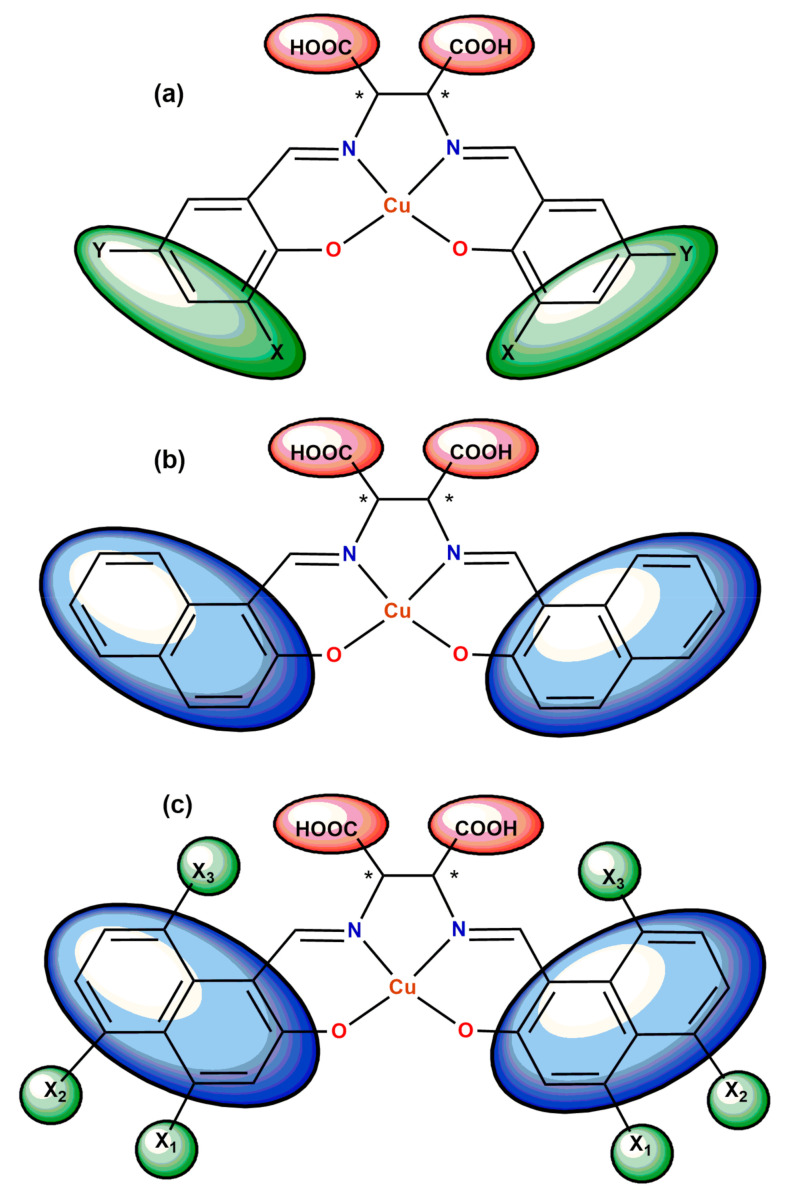
Design of chiral salen-type Schiff base Cu(II) complexes having (**a**) halogen substituents (X, Y) or (**b**) extended π-conjugated system and (**c**) both of these features (* denotes an asymmetric carbon atom) [33,40,41].

In order to improve the power generation efficiency of DSSC the scientists try to broaden the absorption region to the entire visible light and near-infrared region. A successful method seems to be the usage of phthalocyanine, perylene or the like compounds in solar cell materials [37,38,39]. However, there are problems, such as high cost, low absorption strength, dye regeneration due to narrow HOMO-LUMO gap and reduction of the electron injection rate into the semiconductor for example TiO_2_. Therefore, new building blocks based on chiral salen-type Cu (II) complexes with carboxyl groups attached to the ethylenediamine linker were synthesized by Yamane et al., Takahashi et al. and Yamaguchi et al. (Figure 3) [33,40,41]. The carboxylic acid group function was to anchor the organometallic dye on the oxide surface. The complexes absorbed the light at a longer wavelength. The authors studied the band shift of the electronic/CD spectrum due to the effect of the substituent of the ligand of the chiral Schiff base metal complex.

The combination of these both molecular features—halogen substitution at π-conjugated moiety (Figure 3c), resulted in dual purpose materials with possible application as DSSC dyes with enhanced incombustibility of PMMA films [41].

**Figure 4:**
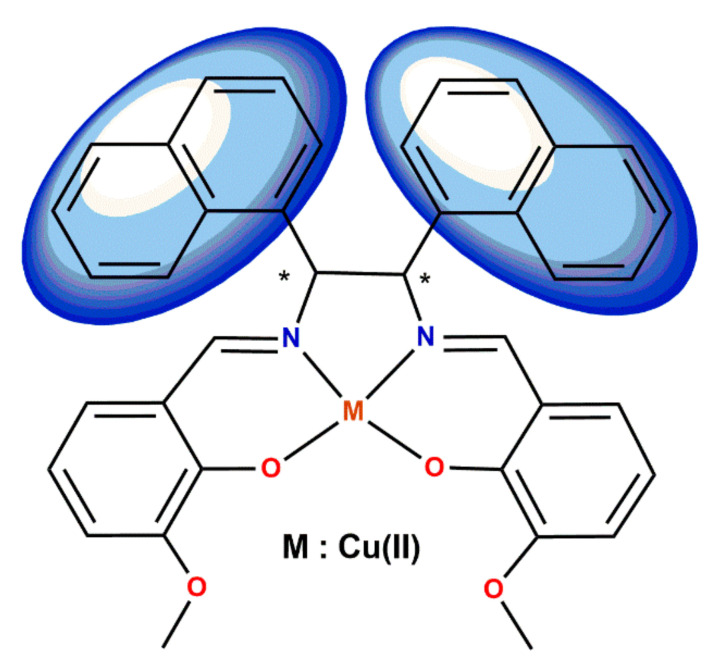
Chiral salen-type Schiff base Cu(II) complexes with π-conjugated ligand (* denotes an asymmetric carbon atom) [42].

On the other hand, Matsuno et al. [42] introduced 1-naphthyl groups at the ethylenediamine bridge instead of carboxy ones to improve the electron transfer properties (Figure 4). This procedure induced a change of dipole moment of TiO_2_ particles due to chemical adsorption, and affected their electronic state.

**Figure 5:**
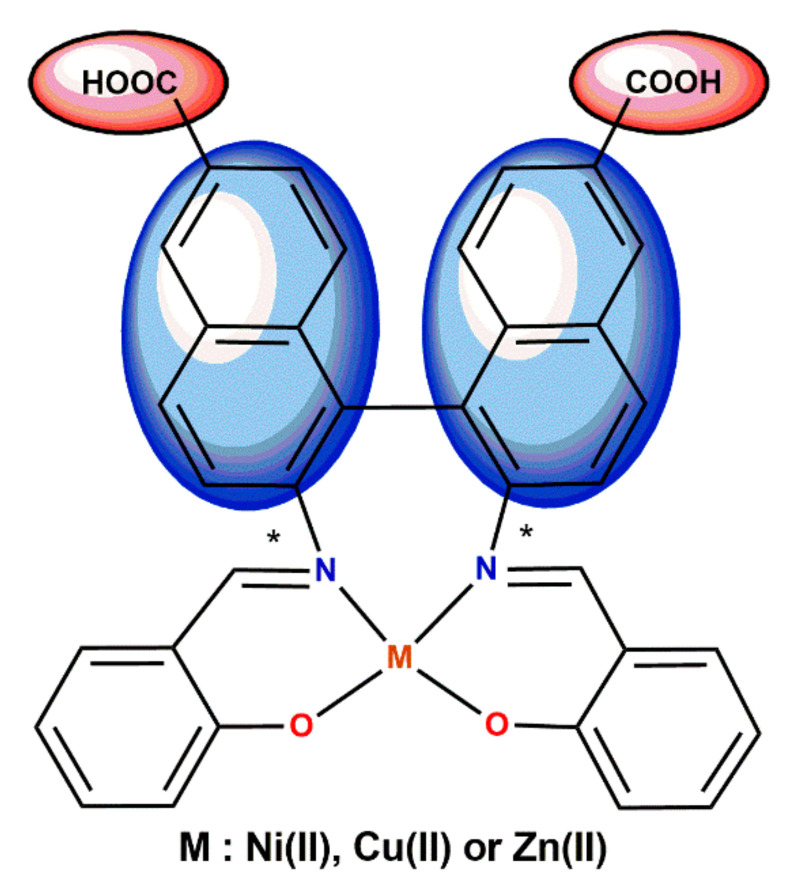
Schiff base complexes with both functionalities: hydrophilic carboxylic groups and π-conjugated systems (* denotes an asymmetric carbon atom) [43].

In turn, Tsaturyan et al. decided to use carboxyl substituted naphthyl moieties (Figure 5) [43]. The advantage of this approach was that the hydrophilic COOH groups increased the adsorption energy, whereas the extended π-conjugated aromatic groups prevented the dye from aggreging on the semiconductor surface.

**Figure 6:**
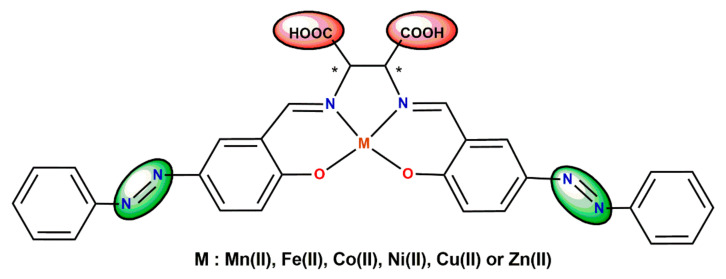
Salen-type transition metal complexes incorporating azo group (* denotes an asymmetric carbon atom) [44,45].

Sato et al. [44,45] have studied absorption wavelength shifts and intramolecular charge transfer in a series of salen-type transition metal complexes incorporating azo group (Figure 6). They discovered that linearly polarized UV light may increase the adsorption amounts of dye complexes onto TiO_2_ due to photo-isomerization and special dye-complex alignment.

**Figure 7:**
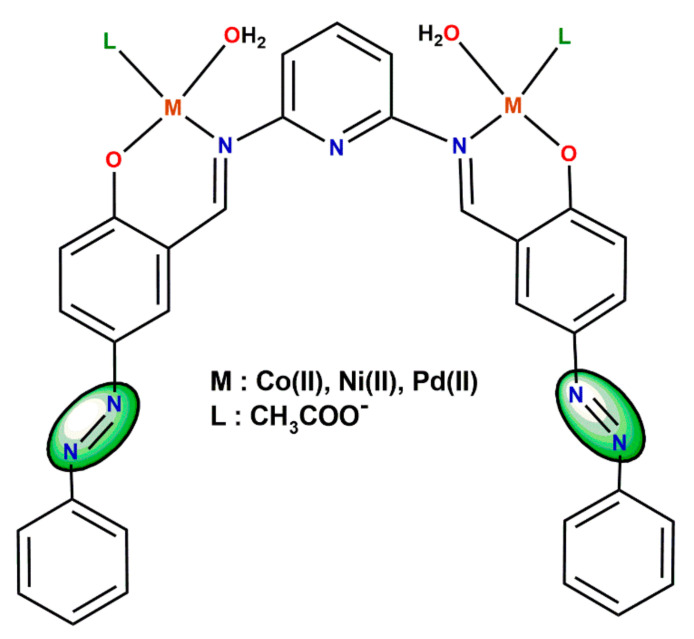
Symmetrical pyridine bridged azo-Schiff base metal complexes for DSSC [46].

Imer et al. [46] developed a different group of symmetrical pyridine bridged azo-Schiff base metal complexes for DSSC (Figure 7). The application of pyridine resulted in a series of nonchiral, dinuclear species, with the solar conversion efficiency reported to be lower than 1%.

## 4. Co-Sensitization Using the Schiff Base Metal Complexes

Co-sensitization is a method for enhancing the performance of DSSC devices by using a combination of two or more photosensitizing dyes to broaden the light-harvesting abilities and to improve the efficiency.

**Figure 8:**
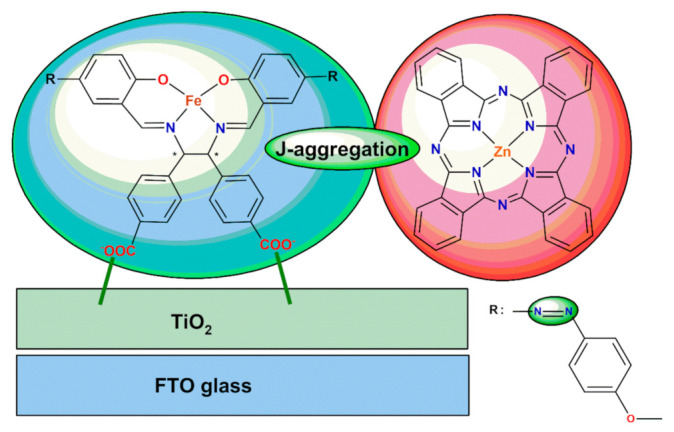
Formation of J-aggregation of Fe(II) and Zn(II) complexes on the surface of TiO_2_ (* denotes an asymmetric carbon atom). Adapted with permission from Ref. [47]. Copyright 2022, Elsevier.

In order to widen the range of absorption wavelength and to enhance the spectral response in photoelectric conversion systems Iwama and Soejima et al. [47] adsorbed two kinds of dyes on TiO_2_ simultaneously—chiral azo-salen Schiff base Fe(II) complex and Zn(II) phthalocyanine one (Figure 8). The purpos e of this attempt was to develop a new photoelectric conversion mechanism using the aggregation interaction of these complexes. The peak of Zn(II) complex in the UV-Vis spectra was observed to be shifted from 343 nm to 346 nm which suggests intermolecular interaction like J-aggregation. The photoelectric effect in the cell incorporating Fe(II) and Zn(II) species was observed in the near infrared region from 900 nm to 1000 nm.

Zhang et al. [48] used the co-sensitization approach for enhancing the overall conversion efficiency of DSSC based on Schiff base transition metal-coordination complexes and a dye N719 deposited on ZnO electrode. They observed not only the extension of the electrode photo-response in the UV region, but also an increase of the intensity in the absorption spectrum.

Dong et al. [49] also studied the co-sensitization with the use of dye N719 and Schiff base transition metal complexes. They reported a luminescence tuning of the hybrid system induced by the structure of the complex. The overall conversion efficiency for the studied material was of ca. 7%, and it was higher than for the devices sensitized only by N719.

## 5. Ligand-Induced Chirality in Chiral Schiff Base Complex @ Nanoparticles Composites

It is known that the adsorption of a chiral substance on an achiral medium or reverse may affect the spectrum of a circular dichroism (CD) of such composites causing an interesting phenomenon of induced circular dichroism (ICD) [50,51,52,53]. Tsutsumi et al. [54,55,56,57,58] observed such effects in hybrid systems of Schiff base metal complexes deposited on nano-inorganic substances (namely metal nanoparticles or semiconductor nanoparticles). They studied the influence of bromination on the molecular dipole moment and the CD behavior.

**Figure 9:**
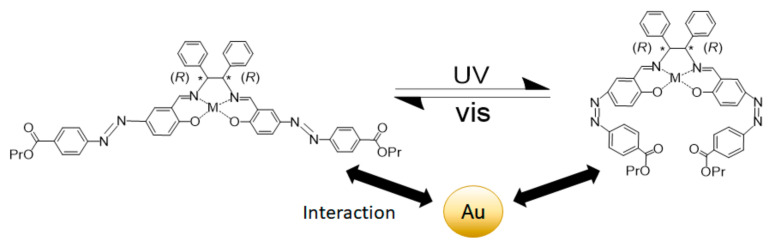
Photoisomerization of azobenzene moiety in chiral Schiff base complexes in the presence of gold nanoparticles (* denotes an asymmetric carbon atom). Reprinted with permission from Ref. [55].

Azobenzene compounds can be aligned by irradiation of linearly polarized UV light due to Weigert effect [59,60,61,62]. In order to interpret the interaction of light and azobenzene compounds, as well as, intermolecular interaction of the photofunctional hybrid materials, Sunaga et al. [55] developed composites of chiral azo-Schiff base complexes @ gold nanoparticles (Figure 9). With the use of TD-DFT calculations and experiments performed with linearly polarized UV light they managed to elucidate the changes in induced circular dichroism spectra by the contributions of the Weigert effect and *cis*-*trans* photoisomerization of the azobenzene molecular fragments.

**Figure 10:**
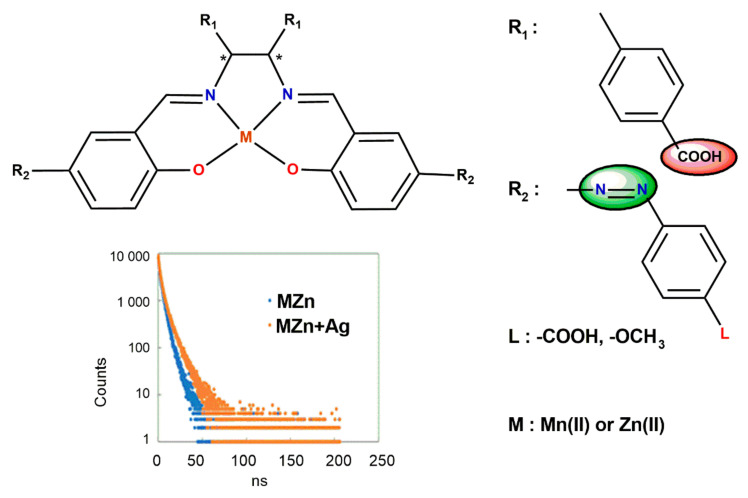
Proposed structure of chiral Schiff base complexes combined with silver nanoparticles and titanium oxide. Long lifetime formed hybrid materials measured with time-resolving luminescence (* denotes an asymmetric carbon atom). Adapted with permission from Ref. [63].

Saiga et al. [63] have prepared hybrid materials of new chiral azo-Schiff base Mn(II) and Zn(II) complexes adsorbed at silver nanoparticles or TiO_2_ (Figure 10). They investigated their chiroptical properties. In order to understand excited state and reaction intermediates during light irradiation to return to the ground state, the time-resolving luminescence measurements were employed. The average fluorescence lifetime of the hybrid materials was higher than that observed for the complexes alone.

## 6. Chiral Schiff Base Complexes @ Polymer Matrix

The combining of photochromic compounds with polymethylmethacrylate films (PMMA) is a well-known technique for fabrication of photoactive, thermoplastic materials frequently used in opto-electronic instruments [64,65,66]. 

**Figure 11:**
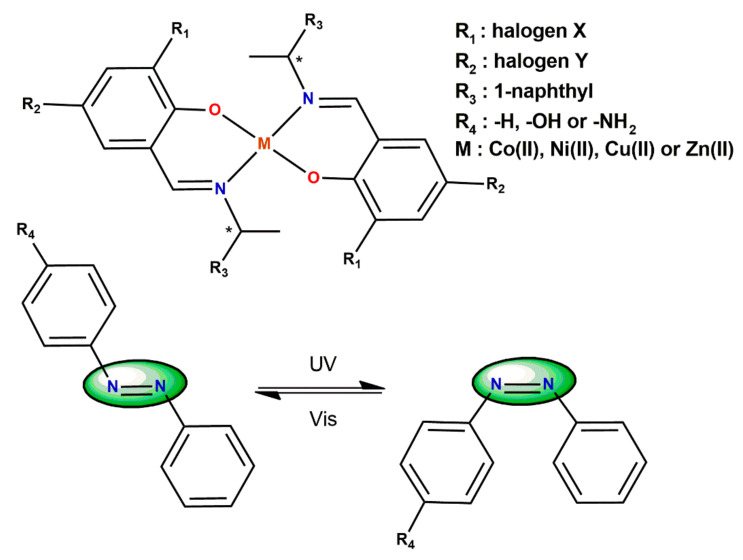
Structures of coordination compounds used by Akitsu and Itoh [67,68] for photoisomerization of azobenzene compounds induced by UV and visible light irradiation (* denotes an asymmetric carbon atom).

Akitsu [67] discussed the mechanism of photo-induced conformational changes of photochromic azo-compounds: azobenzene, 4-hydroxyazobenzene, and 4-aminoazobenzene in the presence of chiral Schiff base metal complexes (Figure 11). Few years later Akitsu and Itoh [68] prepared an organic–inorganic hybrid material built of chiral Schiff base complexes and azobenzene molecules incorporated within a PMMA cast film. The results were analogous, they showed a photo-tuning of optical anisotropy and reversible *cis*-*trans* conformational changes of azobenzene upon UV-Vis light irradiation.

However, in reverse, the presence of azobenzene compound may influence the conformational changes of the chiral ligands. Akitsu and Einaga [69] studied a supramolecular induced solvatochromism in a system composed of 4-hydroxyazobenzene as a photochromic solute and mononuclear nickel (II) complexes incorporating chloro-substituted Schiff base ligands (Figure 11). The distortion of the [NiN_2_O_2_] coordination environment and the changes in the overall dipole moment of the complexes were affected by both the electronic withdrawing Cl-substituents and the steric factors of the ligands. After the UV light irradiation, slight spectral changes in the π–π* region due to a conformational change of the chiral ligands caused by a photochromic solute were observed.

## 7. Chiral Schiff Base Complexes @ Metal-Dendrimer Matrices

Metal-dendrimer nanocomposites are special class of nanomaterials [70]. The dispersion of organic and inorganic species is uniform across the whole material because of pre-organized dendritic macromolecular template. The functionality of such systems may be controlled both by surface modification, as well as, by supramolecular encapsulation.

Akitsu et al. [71] performed some trials to study the hybrid material of chiral Schiff base complexes incorporated into a poly(amido-amine) (PAMAM) dendrimeric template. They synthesized and characterized a hybrid system of chiral Schiff base complexes merged with a metal-dendrimer matrix of Cu-PAMAM G4-NH_2_.

## 8. Applications in the Environmental Protection—The Reduction of Cr(VI) to Cr(III) by TiO_2_ Photocatalyst in the Presence of Schiff Base Transition Metal Complexes

According to the Restriction of Hazardous Substances (RoHS) directive the hexavalent chromium come under limitation to be below 1000 ppm in new electric products, chrome plating, dyes, pigments etc. The disposal of Cr(VI) from the environment is an important issue, and the main method is the reduction of Cr(VI) to less toxic Cr(III) ions. Yoshida et al. [72] have designed and studied a hybrid system for Cr(VI) to Cr(III) reduction reaction. They synthesized four chiral Schiff base Cu(II) complexes having aspartic acid or glutamic acid moieties and combined with TiO_2_. Diphenylcarbazide method indicated that the new hybrid system under irradiating with visible light can reduce Cr(VI) to Cr(III) more efficiently than TiO_2_ severally. Furthermore, even the studied Cu(II) complexes under irradiating light reduced the Cr(VI) ions more effectively than the hybrid systems.

**Figure 12:**
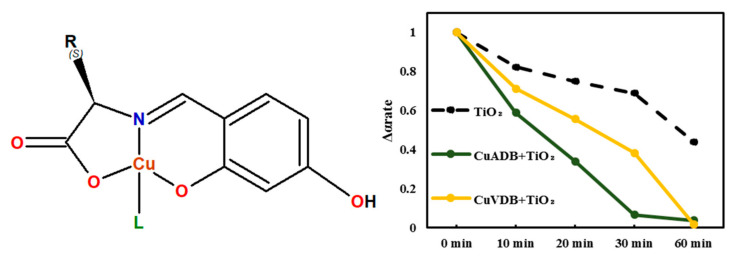
Hydroxy substituted photocatalytic Cu(II) Schiff base complexes studied by Miyagawa et al. (R = -CH_3_ (CuADB), *i*-propyl (CuVDB); L = methanol) and the reduction rate of Cu(II) complexes and TiO_2_ in aqueous solutions after UV light irradiation [73].

Miyagawa et al. [73] worked on the development of homogeneous photocatalysts containing Schiff base Cu(II) complexes for Cr(VI) reduction. They decide to add a polar hydroxyl group to the aromatic ring of the amino acid Schiff base Cu (II) complex. As a result, they reported synthesis and characterization of coordination compounds derived from four amino acids: *L*-alanine, *L*-valine, *L*-phenylalanine, and glycine. The compounds acted as photo-driven reductants in the reduction reaction of Cr(VI) to Cr(III). Upon irradiation with visible and ultraviolet light, the complexes reduced Cr(VI) to Cr(III) more efficiently than methanol and H₂O. The wavelength dependent visible light driven photocatalytic activity of them solely and hybrid systems with TiO₂ in methanol was also investigated (Figure 12).

**Figure 13:**
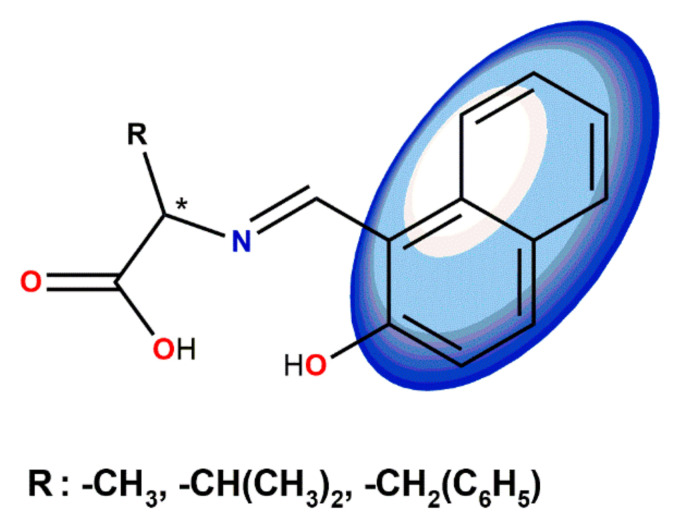
Amino acid Schiff base ligands with an extended π-conjugated system (* denotes an asymmetric carbon atom) [74,75].

Nakagame et al. [74] studied Cu(II) Schiff base complexes with ligands having an extended π-conjugated system on the aldehyde side (Figure 13). As a result, they obtained polymeric coordination compounds with carboxylate groups serving as bridges. Both complexes, as well as the complex @ TiO_2_ composites, showed higher photocatalytic activity in a methanolic solution than the titanium oxide alone. The concentration of Cr(VI) was measured by the diphenylcarbazide method. The incorporation of π-conjugated system resulted in a red shift of the wavelength in the absorption spectra. The electrochemical analysis showed that the Cr(VI)/Cr(III) reduction rate depended on the redox potential of the Schiff base copper complex.

## 9. Applications in Cosmetology—Schiff Base Complexes as Sunscreens

The photoactive properties of some materials may be used in sunscreens to protect the skin from a damage. Apart from the UVA light (up to ca. 400 nm), the IR radiation is also believed to be harmful to the skin, especially by destroying the collagen fibers. Therefore, Onami et al. [75] performed studies on Zn(II) Schiff base complexes with a naphthyl moiety (Figure 13) to examine their ability to absorb in the region of infrared radiation by an infrared free electron laser method (IR-FEL). The motivation of the project was to determine if the new material may protect the human serum albumin.

## 10. Real-Time Visualization of Cellular Processes—QDs Doped with Schiff Base Complex for Real-Time Visualization of Cell Membrane Damages

Quantum dots (QDs) are one of the newest members of nanomaterials. Due to the nano size their optical properties are exceptional compared to other photofunctional materials. However, one of the limitations of semiconducting representatives is their hydrophobicity and low solubility in water. The doping with other substances enhances their biocompatibility and broadens the range of application in real-time visualization of cellular processes, among others [76,77,78,79].

Moulick et al. [80] reported an interesting application of Schiff base Gd(III) complex for modification of CdTe quantum dots. They used the fluorescent properties of the hybrid material together with its ability to interact preferentially with a specific fragment of protein building the cell membrane to visualize the cell membrane damages in real-time. This approach has the advantage of higher resolution and shorter response time compared to organic dyes. It gives opportunity to apply such materials in examination of cellular membranes, host–pathogen interactions or cell-penetrating drug mechanisms of action.

## 11. Schiff Base Complexes @ CdTe QDs as Cytocompatible Fluorophores for Bio-Labeling

Buchtelova et al. [81] used the Schiff base Gd(III), Tb(III) and Yb(III) complexes for doping of CdTe QDs to decrease their cytotoxicity. The reported composite was water-soluble, cytocompatible and its fluorescence properties were stable, what predestinates this kind of material to be applied successfully in bio-labeling.

## 12. Light Activated Prodrug Applications

**Figure 14:**
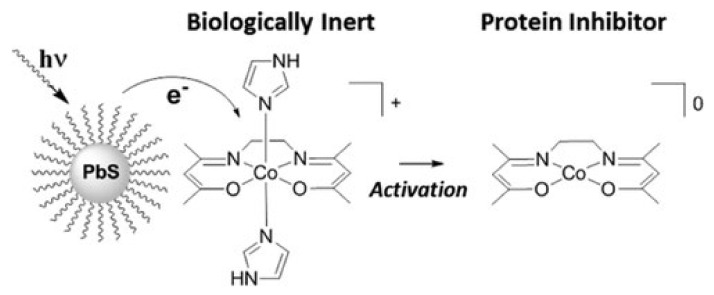
Photoinduced electron transfer (PET) process between Co(III) Schiff base complex and colloidal PbS quantum dots. Reprinted with permission from Ref. [86]. Copyright 2013, American Chemical Society.

Schiff base metal complexes exhibit a wide range of biological activities and often such systems are used to mimic naturally occurring catalytic active centers [82,83,84,85]. Peterson et al. described a light induced activation of a protein inhibitor by a hybrid material of Schiff-base complexes @ colloidal PbS QDs [86]. The pivotal factor was the cooperation between Co(III) Schiff base complex and colloidal PbS quantum dots Figure 14. The activation of the biologically inert coordination compound was possible due to the photoinduced electron transfer (PET).

Later Holbrook et al. presented a mechanism of light activation of an analogous Co(III) Schiff base complex. They used this coordination compound instead of a covalently bonded Ru(II) bipyridyl complex. In both cases the photoinduced electron transfer occurred easily [87]. These experiments show that the Schiff base complex @ PbS QDs hybrid materials may be efficiently used for light activated prodrug applications, and may replace covalently bonded species [88].

## 13. Summary

In this review, we presented a brief overview of some current directions of applications of photofunctions of hybrid systems based on Schiff base metal complexes and metal or semiconductor (nano)materials.

We tried to line out the most interesting directions of possible applications of these photoactive hybrid materials including light-energy conversion systems, dye sensitized solar cells, photocatalysis, or medical applications in bio-labeling, or light triggered prodrug activation.

These composite materials provide new catalytic and optical properties not observed for each component separately. The synergistic effects noticed for such systems may help in controlling the absorption-emission wavelength ranges in new materials just by the modification of the ligand molecules in the Schiff base complexes. This approach has the advantage due to the ease of designing and obtaining of new azomethine compounds. The tuning effect may be achieved in various hybrid systems including metal or semiconductor nanoparticles, as well as, in polymeric or metal-dendrimer matrices.

The ease of incorporation of Schiff base metal complexes into various hybrid systems together with the developing research area of photofunctional nanomaterials will bring some new solutions in the near future. This research area needs the theoretical background to be explored in detail to help in designing a priori new materials with desirable properties. The development of new hybrid systems may be carried out via two routes. First, the Schiff base ligands may be modified to tune the photofunctions, to broaden the absorption range and to deliver better sensitizers for composite materials. The second promising direction seems to be the usage of other types of components or matrices for such systems. An example may be the non-toxic carbon quantum dots, which may provide devices for medical or environmentally friendly nanotechnological applications.

## Abbreviations

CD	Circular Dichroism
IR	Infrared
IR-FEL	Infrared Free Electron Laser
PAMAM	poly(amido-amine) (PAMAM) dendrimer
PET	Photoinduced Electron Transfer
QDs	quantum dots

## References

[1] Yang X., Wang D. (2018). Photocatalysis: From Fundamental Principles to Materials and Applications. ACS Appl. Energy Mater..

[2] Melchionna M., Fornasiero P. (2020). Updates on the Roadmap for Photocatalysis. ACS Catal..

[3] Lu K.-Q., Quan Q., Zhang N., Xu Y.-J. (2016). Multifarious roles of carbon quantum dots in heterogeneous photocatalysis. J. Energy Chem..

[4] Sharma S., Umar A., Sood S., Mehta S.K., Kansal S.K. (2018). Photoluminescent C-dots: An overview on the recent development in the synthesis, physiochemical properties and potential applications. J. Alloys Compd..

[5] Pal A., Sk M.P., Chattopadhyay A. (2020). Recent advances in crystalline carbon dots for superior application potential. Mater. Adv..

[6] Hazaraimi M.H., Goh P.S., Lau W.J., Ismail A.F., Wu Z., Subramaniam M.N., Lim J.W., Kanakaraju D. (2022). The state-of-the-art development of photocatalysts for the degradation of persistent herbicides in wastewater. Sci. Total Environ..

[7] Liu X., Hamon J.-R. (2019). Recent developments in penta-, hexa- and heptadentate Schiff base ligands and their metal complexes. Coord. Chem. Rev..

[8] Mehrabian M., Dalir S., Mahmoudi G., Miroslaw B., Babashkina M.G., Dektereva A.V., Safin D.A. (2019). A Highly Stable All-Inorganic CsPbBr_3_ Perovskite Solar Cell. Eur. J. Inorg. Chem..

[9] Hu H., Wang L., Wang L., Li L., Feng S. (2020). Imine-functionalized polysiloxanes for supramolecular elastomers with tunable mechanical properties. Polym. Chem..

[10] González D.M., Hernández L.A., Oyarce J., Alfaro A., Novoa N., Cisterna J., Brito I., Carrillo D., Manzur C. (2021). A new and efficient high-performance electrochemical glucose sensor based on a metallopolymer derived from a cobaltate (III) Schiff base complex. Synth. Met..

[11] Li N., Kang G., Liu H., Li M., Qiu W., Wang Q., Liu L., Yu J., Li B., Li F. (2022). Hybrid nanoparticles based on novel Schiff Base for durable flame retardant and antibacterial properties. Compos. Part B Eng..

[12] Deng B.B., Cheng T.T., Hu Y.T., Cheng S.P., Huang C.R., Yu H., Wang Z.X. (2022). The first salicylaldehyde Schiff base organic-inorganic hybrid lead iodide perovskite ferroelectric. Chem. Commun..

[13] Afrin Dalia S., Farhana Afsan B., Md Saddam Hossain B., Nuruzzaman Khan M., Md Kudrat-E-Zahan B., Md Mahasin Ali B., Md Kudrat-E-Zahan C., Afsan F., Saddam Hossain M., Zakaria C. (2018). A short review on chemistry of schiff base metal complexes and their catalytic application. Int. J. Chem. Stud..

[14] Radecka-Paryzek W. (2009). Self-assembly in schiff base lanthanide complexes—From supramolecular dimers to coordination polymers. Can. J. Chem..

[15] Liu X., Manzur C., Novoa N., Celedón S., Carrillo D., Hamon J.-R.R. (2018). Multidentate unsymmetrically-substituted Schiff bases and their metal complexes: Synthesis, functional materials properties, and applications to catalysis. Coord. Chem. Rev..

[16] Tsantis S.T., Tzimopoulos D.I., Holynska M., Perlepes S.P. (2020). Oligonuclear Actinoid Complexes with Schiff Bases as Ligands—Older Achievements and Recent Progress. Int. J. Mol. Sci..

[17] Miroslaw B. (2020). Homo-and hetero-oligonuclear complexes of platinum group metals (PGM) coordinated by imine Schiff base ligands. Int. J. Mol. Sci..

[18] Sztanke K., Maziarka A., Osinka A., Sztanke M. (2013). An insight into synthetic Schiff bases revealing antiproliferative activities in vitro. Bioorganic Med. Chem..

[19] Raczuk E., Dmochowska B., Samaszko-Fiertek J., Madaj J. (2022). Different Schiff Bases—Structure, Importance and Classification. Molecules.

[20] Catalano A., Sinicropi M.S., Iacopetta D., Ceramella J., Mariconda A., Rosano C., Scali E., Saturnino C., Longo P. (2021). A review on the advancements in the field of metal complexes with schiff bases as antiproliferative agents. Appl. Sci..

[21] Takeshita Y., Takakura K., Akitsu T. (2015). Multifunctional composites of chiral valine derivative Schiff base Cu(II) complexes and TiO2. Int. J. Mol. Sci..

[22] Yamamoto S., Akitsu T. (2011). Fluorescence detection by photochromic dye of photoinduced electron transfer reactions between lysine and methionine derivative Schiff base copper(II) complexes and titanium oxide. Asian Chem. Lett..

[23] Kurata M., Yoshida N., Fukunaga S., Akitsu T. (2013). Proposed Mechanism of Photo-Induced Reactions of Chiral Threonine Schiff Base Cu(II) Complexes with Imidazole by TiO 2. Contemp. Eng. Sci..

[24] Takeshita Y., Nogami A., Akitsu T. (2014). UV light-induced reaction of chiral arginine derivative-Schiff base Cu(II) complexes and TiO_2_. World Sci. Echo.

[25] Takeshita Y., Akitsu T. (2015). Multi-Functional Composites and Substituent Effects of Chiral Asparagine-Schiff Base Cu(II) Complexes and Titanium(IV) Oxide. Pure Appl. Chem. Sci..

[26] Nishizuru H., Kimura N., Akitsu T. (2012). Photo-induced reduction of hybrid systems of phenylalanine and other derivatives of Schiff base Cu(II) complexes and titanium(IV) oxide. Asian Chem. Lett..

[27] Nakayama T., Minemoto M., Nishizuru H., Akitsu T. (2011). Spectroelectrochemistry of photoinduced electron transfer reactions between leucine and serine derivative Schiff base copper(II) complexes and titanium Oxide. Asian Chem. Lett..

[28] Yoshida N., Akitsu T. (2013). Reaction of Hybrid Systems Composed of Cu(II) Complexes Having Chiral Schiff Base Amino-Acid Ester Derivatives and TiO2. Integrating Approach to Photofunctional Hybrid Materials for Energy and the Environment.

[29] Gong J., Sumathy K., Qiao Q., Zhou Z. (2017). Review on dye-sensitized solar cells (DSSCs): Advanced techniques and research trends. Renew. Sustain. Energy Rev..

[30] Francis O.I., Ikenna A., Francis O.I., Ikenna A. (2021). Review of Dye-Sensitized Solar Cell (DSSCs) Development. Nat. Sci..

[31] Baby R., Nixon P.D., Kumar N.M., Subathra M.S.P., Ananthi N. (2022). A comprehensive review of dye-sensitized solar cell optimal fabrication conditions, natural dye selection, and application-based future perspectives. Environ. Sci. Pollut. Res. Int..

[32] Kokkonen M., Talebi P., Zhou J., Asgari S., Soomro S.A., Elsehrawy F., Halme J., Ahmad S., Hagfeldt A., Hashmi S.G. (2021). Advanced research trends in dye-sensitized solar cells. J. Mater. Chem. A.

[33] Yamaguchi M., Tsunoda Y., Tanaka S., Haraguchi T., Sugiyama M., Noor S., Akitsu T. (2017). Orbital and molecular design of new naphthyl-salen type transition metal complexes toward DSSC dyes. J. Indian Chem. Soc..

[34] Hagfeldt A., Boschloo G., Sun L., Kloo L., Pettersson H. (2010). Dye-sensitized solar cells. Chem. Rev..

[35] Mahadevi P., Sumathi S. (2020). Mini review on the performance of Schiff base and their metal complexes as photosensitizers in dye-sensitized solar cells. Synth. Commun..

[36] Yamaguchi M., Takahashi K., Akitsu T. (2016). Molecular design through TD-DFT calculation of chiral salen Cu II complexes toward NIR absorption for DSSC. J. Indian Chem. Soc.

[37] Cappel U.B., Karlsson M.H., Pschirer N.G., Eickemeyer F., Schöneboom J., Erk P., Boschloo G., Hagfeldt A. (2009). A broadly absorbing perylene dye for solid-state dye-sensitized solar cells. J. Phys. Chem. C.

[38] Milan R., Selopal G.S., Cavazzini M., Orlandi S., Boaretto R., Caramori S., Concina I., Pozzi G. (2017). Dye-sensitized solar cells based on a push-pull zinc phthalocyanine bearing diphenylamine donor groups: Computational predictions face experimental reality. Sci. Rep..

[39] Ragoussi M.E., Ince M., Torres T. (2013). Recent Advances in Phthalocyanine-Based Sensitizers for Dye-Sensitized Solar Cells. European J. Org. Chem..

[40] Yamane S., Hiyoshi Y., Tanaka S., Ikenomoto S., Numata T., Takakura K., Haraguchi T., Palafox M.A., Hara M., Sugiyama M. (2018). Substituent Effect of Chiraldiphenyl Salen Metal (M = Fe(II), Co(II), Ni(II), Cu(II), Zn(II)) Complexes for New Conceptual DSSC Dyes. J. Chem. Chem. Eng..

[41] Takahashi K., Tanaka S., Yamaguchi M., Tsunoda Y., Akitsu T., Sugiyama M., Soni R.K., Moon D. (2017). Dual purpose Br-containing Schiff base Cu(II) complexes for DSSC dyes and polymer flame retardants. J. Korean Chem. Soc..

[42] Matsuno M., Noor S., Numata T., Haraguchi T., Akitsu T., Hara M. (2017). Synthesis and structural characterization of new [CuII-TiO2] composites from CuII-salen as precursors. J. Indian Chem. Soc..

[43] Tsaturyan A., Machida Y., Akitsu T., Gozhikova I., Shcherbakov I. (2018). Binaphthyl-containing Schiff base complexes with carboxyl groups for dye sensitized solar cell: An experimental and theoretical study. J. Mol. Struct..

[44] Tanaka S., Sato H., Ishida Y., Deng Y., Haraguchi T., Akitsu T., Sugiyama M., Hara M., Moon D. (2018). Photo-Control of Adsorption of Dye Metal Complexes Incorporating Chiral Schiff Base Ligands Containing Azo-Groups on TiO_2_. J. Korean Chem. Soc..

[45] Sato H., Beppu I., Haraguchi T., Akitsu T., Parida R., Giri S., Roymahapatra G., Hubert Joe I. (2018). Optical properties of chiral Schiff base MnII, CoII, NiII complexes having azobenzene. J. Indian Chem. Soc..

[46] Gencer Imer A., Syan R.H.B., Gülcan M., Ocak Y.S., Tombak A. (2018). The novel pyridine based symmetrical Schiff base ligand and its transition metal complexes: Synthesis, spectral definitions and application in dye sensitized solar cells (DSSCs). J. Mater. Sci. Mater. Electron..

[47] Akitsu T., Iwama J. (2022). Salen-type metal complexes based on structural database of X-ray crystallography. Computational and Data-Driven Chemistry Using Artificial Intelligence.

[48] Zhang L., Yang Y., Fan R., Wang P., Li L. (2012). Enhance the performances of dye-sensitized solar cell by a new type of sensitizer to co-sensitize zinc oxide photoelectrode with ruthenium complex. Dye. Pigment..

[49] Dong Y.W., Fan R.Q., Wang P., Wei L.G., Wang X.M., Zhang H.J., Gao S., Yang Y.L., Wang Y.L. (2015). Synthesis and characterization of substituted Schiff-base ligands and their d10 metal complexes: Structure-induced luminescence tuning behaviors and applications in co-sensitized solar cells. Dalt. Trans..

[50] Kuznetsova V., Gromova Y., Martinez-Carmona M., Purcell-Milton F., Ushakova E., Cherevkov S., Maslov V., Gun’Ko Y.K. (2020). Ligand-induced chirality and optical activity in semiconductor nanocrystals: Theory and applications. Nanophotonics.

[51] Allenmark S. (2003). Induced circular dichroism by chiral molecular interaction. Chirality.

[52] Gawroński J., Grajewski J. (2003). The significance of induced circular dichroism. Org. Lett..

[53] Akitsu T., Kim S., Nakane D. (2021). Induced CD on metal nanoclusters or other materials by chiral Schiff base metal complexes. Trends Photochem. Photobiol..

[54] Tsutsumi Y., Sunaga N., Haraguchi T., Akitsu T. (2017). Induced CD from chiral Schiff base metal complexes involving azo-dye groups to gold nanoparticles. J. Indian Chem. Soc..

[55] Sunaga N., Haraguchi T., Akitsu T. (2019). Orientation of Chiral Schiff Base Metal Complexes Involving Azo-Groups for Induced CD on Gold Nanoparticles by Polarized UV Light Irradiation. Symmetry.

[56] Oshima M., Matsuno M., Yuki T., Nobumitsu S., Haraguchi T., Akitsu T., Oshima M., Matsuno M., Yuki T., Nobumitsu S. (2017). Synthesis of Chiral Schiff Base Metal Complex Inducing CD and Elucidation of Structure of Adsorption on Surface of Gold Nanoparticles. Int. J. Org. Chem..

[57] Kimura N., Nishizuru H., Aritake Y., Akitsu T. (2013). Observation of reciprocal induced CD between colloidal gold nanoparticles and chiral Schiff base Zn(II) complexes with parallel dipole moments. J. Chem. Chem. Eng..

[58] Aritake Y., Nakayama T., Nishizuru H., Akitsu T. (2011). Observation of induced CD on CdSe nanoparticles from chiral Schiff base Ni(II), Cu(II), Zn(II) complexes. Inorg. Chem. Commun..

[59] Bazadze M.A., Ebralidze N.A., Ebralidze T.D. (2002). Weigert’s effect mechanism observed in dyes. Appl. Opt..

[60] Petrova S.S., Chichinadze N.M., Shaverdova V.G. (2005). Kinetics of the Weigert effect in azo dyes embedded in polymeric matrices with different activities. Tech. Phys..

[61] Iftime G., Labarthet F.L., Natansohn A., Rochon P. (2000). Control of Chirality of an Azobenzene Liquid Crystalline Polymer with Circularly Polarized Light. J. Am. Chem. Soc..

[62] Yang G., Zhang S., Hu J., Fujiki M., Zou G. (2019). The chirality induction and modulation of polymers by circularly polarized light. Symmetry (Basel)..

[63] Saiga K., Haraguchi T., Kitahama Y., Hosokai T., Matsuzaki H., Moon D., Sugiyama M., Hara M., Akitsu T., Saiga K. (2021). Optical Properties of Chiral Azo-Schiff Base Mn(II) and Zn(II) Complexes with Silver Nanoparticles. J. Mater. Sci. Chem. Eng..

[64] Albeladi H.K., Al-Romaizan A.N., Hussein M.A. (2017). Role of cross-linking process on the performance of PMMA. Int. J. Biosens. Bioelectron..

[65] Zafar M.S. (2020). Prosthodontic applications of polymethyl methacrylate (PMMA): An update. Polymers.

[66] Rahman F., Carbaugh D.J., Wright J.T., Rajan P., Pandya S.G., Kaya S. (2020). A review of polymethyl methacrylate (PMMA) as a versatile lithographic resist—With emphasis on UV exposure. Microelectron. Eng..

[67] Akitsu T. (2007). Photofunctional supramolecular solution systems of chiral Schiff base nickel(II), copper(II), and zinc(II) complexes and photochromic azobenzenes. Polyhedron.

[68] Akitsu T., Itoh T. (2010). Polarized spectroscopy of hybrid materials of chiral Schiff base cobalt(II), nickel(II), copper(II), and zinc(II) complexes and photochromic azobenzenes in PMMA films. Polyhedron.

[69] Akitsu T., Einaga Y. (2005). Synthesis, crystal structures and electronic properties of Schiff base nickel (II) complexes: Towards solvatochromism induced by a photochromic solute. Polyhedron.

[70] Balogh L., Laverdure K.S., Gido S.P., Mott A.G., Miller M.J., Ketchel B.P., Tomalia D.A. (1999). Dendrimer-Metal Nanocomposites. MRS Online Proc. Libr..

[71] Akitsu T., Yamaguchi J., Uchida N., Aritake Y. (2009). The studies of conditions for inducing chirality to Cu(II) complexes by chiral Zn(II) and Ni(II) complexes with Schiff base. Res. Lett. Mater. Sci..

[72] Yoshida N., Tsaturyan A., Akitsu T., Tsunoda Y., Shcherbakov I. (2017). Wavelengths dependence of photo-induced reactions of hybrid systems of Schiff base Cu(II) complexes and TiO2 for Cr(VI) reduction. Russ. Chem. Bull..

[73] Miyagawa Y., Tsatsuryan A., Haraguchi T., Shcherbakov I., Akitsu T. (2020). Photochemical reduction of Cr(VI) compounds by amino acid Schiff base copper complexes with a hydroxyl group and titanium oxide composites in aqueous solutions. New J. Chem..

[74] Nakagame R., Tsaturyan A., Haraguchi T., Pimonova Y., Lastovina T., Akitsu T., Shcherbakov I. (2019). Photochemical reaction of amino acid Schiff base derived Cu complexes with extended π-system and their titanium oxide composites. Inorg. Chim. Acta.

[75] Onami Y., Koya R., Kawasaki T., Aizawa H., Nakagame R., Miyagawa Y., Haraguchi T., Akitsu T., Tsukiyama K., Palafox M.A. (2019). Investigation by DFT Methods of the Damage of Human Serum Albumin Including Amino Acid Derivative Schiff Base Zn(II) Complexes by IR-FEL Irradiation. Int. J. Mol. Sci..

[76] Zhang Y.Q., Ma D.K., Zhang Y.G., Chen W., Huang S.M. (2013). N-doped carbon quantum dots for TiO2-based photocatalysts and dye-sensitized solar cells. Nano Energy.

[77] Bera D., Qian L., Tseng T.K., Holloway P.H. (2010). Quantum dots and their multimodal applications: A review. Materials.

[78] Jain K.K. (2007). Applications of nanobiotechnology in clinical diagnostics. Clin. Chem..

[79] Pisanic T.R., Zhang Y., Wang T.H. (2014). Quantum dots in diagnostics and detection: Principles and paradigms. Analyst.

[80] Moulick A., Heger Z., Milosavljevic V., Richtera L., Barroso-Flores J., Merlos Rodrigo M.A., Buchtelova H., Podgajny R., Hynek D., Kopel P. (2018). Real-Time Visualization of Cell Membrane Damage Using Gadolinium-Schiff Base Complex-Doped Quantum Dots. ACS Appl. Mater. Interfaces.

[81] Buchtelova H., Strmiska V., Skubalova Z., Dostalova S., Michalek P., Krizkova S., Hynek D., Kalina L., Richtera L., Moulick A. (2018). Improving cytocompatibility of CdTe quantum dots by Schiff-base-coordinated lanthanides surface doping. J. Nanobiotechnology.

[82] Pervaiz M., Munir A., Riaz A., Saeed Z., Younas U., Imran M., Ullah S., Bashir R., Rashid A., Adnan A. (2022). Review article-Amalgamation, scrutinizing, and biological evaluation of the antimicrobial aptitude of thiosemicarbazide Schiff bases derivatives metal complexes. Inorg. Chem. Commun..

[83] De Fátima Â., Pereira C de P., Olímpio C.R.S.D.G., de Freitas Oliveira B.G., Franco L.L., da Silva P.H.C. (2018). Schiff bases and their metal complexes as urease inhibitors—A brief review. J. Adv. Res..

[84] Ritter E., Przybylski P., Brzezinski B., Bartl F. (2009). Schiff Bases in Biological Systems. Curr. Org. Chem..

[85] Abu-Dief A.M., Mohamed I.M.A.A. (2015). A review on versatile applications of transition metal complexes incorporating Schiff bases. Beni-Suef Univ. J. Basic Appl. Sci..

[86] Peterson M.D., Holbrook R.J., Meade T.J., Weiss E.A. (2013). Photoinduced electron transfer from PbS quantum dots to cobalt(III) schiff base complexes: Light activation of a protein inhibitor. J. Am. Chem. Soc..

[87] Holbrook R.J., Weinberg D.J., Peterson M.D., Weiss E.A., Meade T.J. (2015). Light-activated protein inhibition through photoinduced electron transfer of a ruthenium(II)-cobalt(III) bimetallic complex. J. Am. Chem. Soc..

[88] Renfrew A. (2018). Selective Activation of Transition-Metal Complexes in Medicine. Encycl. Inorg. Bioinorg. Chem..

